# Comparative Genomics of Eight *Fusarium graminearum* Strains with Contrasting Aggressiveness Reveals an Expanded Open Pangenome and Extended Effector Content Signatures

**DOI:** 10.3390/ijms22126257

**Published:** 2021-06-10

**Authors:** Tarek Alouane, Hélène Rimbert, Jörg Bormann, Gisela A. González-Montiel, Sandra Loesgen, Wilhelm Schäfer, Michael Freitag, Thierry Langin, Ludovic Bonhomme

**Affiliations:** 1 Diversité, Ecophysiologie des Céréales, Université Clermont Auvergne, INRAE, UMR 1095 Génétique, 63000 Clermont-Ferrand, France; alouane.tarek@gmail.com (T.A.); helene.rimbert@inrae.fr (H.R.); 2 Molekulare Phytopathologie, Institut für Pflanzenwissenschaften und Mikrobiologie, Universität Hamburg, 22609 Hamburg, Germany; bormannj@gmail.com (J.B.); wilhelm.schaefer@gmail.com (W.S.); 3 Department of Chemistry, Oregon State University, Corvallis, OR 97331, USA; gonzalgi@oregonstate.edu (G.A.G.-M.); sandra.loesgen@whitney.ufl.edu (S.L.); 4 Whitney Laboratory for Marine Bioscience, Department of Chemistry, University of Florida, St. Augustine, FL 32080, USA; 5 Department of Biochemistry and Biophysics, Oregon State University, Corvallis, OR 97331, USA; Michael.Freitag@oregonstate.edu

**Keywords:** *Fusarium graminearum*, Fusarium head blight, pangenome, secreted protein clusters, proteinaceous effectors, aggressiveness, intra-species genomic diversity

## Abstract

*Fusarium graminearum*, the primary cause of Fusarium head blight (FHB) in small-grain cereals, demonstrates remarkably variable levels of aggressiveness in its host, producing different infection dynamics and contrasted symptom severity. While the secreted proteins, including effectors, are thought to be one of the essential components of aggressiveness, our knowledge of the intra-species genomic diversity of *F. graminearum* is still limited. In this work, we sequenced eight European *F. graminearum* strains of contrasting aggressiveness to characterize their respective genome structure, their gene content and to delineate their specificities. By combining the available sequences of 12 other *F. graminearum* strains, we outlined a reference pangenome that expands the repertoire of the known genes in the reference PH-1 genome by 32%, including nearly 21,000 non-redundant sequences and gathering a common base of 9250 conserved core-genes. More than 1000 genes with high non-synonymous mutation rates may be under diverse selection, especially regarding the trichothecene biosynthesis gene cluster. About 900 secreted protein clusters (SPCs) have been described. Mostly localized in the fast sub-genome of *F. graminearum* supposed to evolve rapidly to promote adaptation and rapid responses to the host’s infection, these SPCs gather a range of putative proteinaceous effectors systematically found in the core secretome, with the chloroplast and the plant nucleus as the main predicted targets in the host cell. This work describes new knowledge on the intra-species diversity in *F. graminearum* and emphasizes putative determinants of aggressiveness, providing a wealth of new candidate genes potentially involved in the Fusarium head blight disease.

## 1. Introduction

Fusarium head blight (FHB), caused mainly by the Ascomycete fungus *Fusarium graminearum*, is a devastating disease of small grain cereals, and in particular wheat, which results in yield losses and reduced grain quality [1,2]. The main consequence of FHB is the accumulation of mycotoxins, such as trichothecenes deoxynivalenol (DON) and its derivatives, in the grain, which poses serious health risks, including immunological and teratogenic effects in humans [3,4,5]. *F. graminearum* has been defined as a member of the *F. graminearum* species complex (FGSC), comprising at least 16 phylogenetically distinct species found worldwide [6,7]. *F. graminearum* is the predominant species associated with FHB in North and South America [8,9,10,11,12], in China [13,14] and in most European countries, e.g., France [15,16,17], Germany [18,19,20] and Italy [21,22,23].

Genome decoding is the first step to decipher the range of molecular mechanisms driving diversification, genetic variability and host–pathogen interactions. In *F. graminearum*, the genome size reaches 36–38 Mb, with about 14,000 identified gene models and a bipartite “two-speed genome” architecture [24,25,26,27,28,29]. Horizontal gene transfer events have already been suggested between *F. graminearum* and other fungi [30]. However, our knowledge of the complete gene set of *F. graminearum* still remains incomplete, because a single reference genome does not reflect the genetic variability within a species. Genetic variants, new haplotypes, change total genetic content and thus expand the number of unique gene models to the pangenome concept [31,32,33,34]. Hence, the pangenome includes (i) the core genome, i.e., genes conserved in all isolates or strains, and (ii) the dispensable or accessory genome, i.e., genes shared by only a subset of isolates or strains, or even specific to a single genome [35]. Such compendia offer unprecedented opportunities to discover new genes, explore genomic diversity and improve our knowledge of the evolutionary forces that shape the organization and dynamics of the genomes. In the *F. graminearum* species, designing and analyzing its full gene repertoire and defining its core gene set could provide new knowledge on the genetic determinants of plant–pathogen interactions, further discovering original ways of deciphering the molecular components of pathogenicity.

During the course of infection, the pathogen synthesizes a complex arsenal of secreted proteins, particularly proteinaceous effectors able to deregulate the main plant biological processes, such as the immune system, in order to facilitate the pathogen development in the plant host tissues [36,37]. These proteinaceous effectors are secreted and maintained either in the apoplast, or translocated into plant cells to target specific intracellular processes [38]. Known secreted effectors include protease inhibitors that override host proteases and active carbohydrate enzymes (CAZymes) that can weaken the plant cell walls [39,40,41,42]. Based on the first sequenced genome of *F. graminearum* (strain PH-1 [24]), Brown et al. [43] predicted a secretome of 574 proteins. The contrasting aggressiveness of *F. graminearum* strains on wheat and their ability to synthesize a quantitatively variable “effectome” [17] strongly suggest that the genetic determinant of pathogenicity, such as the effector content and sequence, are not similar. However, our knowledge of the profile and functional characteristics of the secretomes of strains harboring contrasting aggressiveness remains poorly documented and still requires to be connected with phenotype data.

Along with proteinaceous effectors, *F. graminearum* also has the ability to produce a set of secondary metabolites. Some of them are known to be involved in virulence, achieving effector-like impacts in the host plants [44,45,46,47]. Secondary metabolites are produced by proteins encoded by genes found in biosynthetic gene clusters (BGCs) [30,48], within which the expression of a few genes may be sufficient to the biosynthesis of a particular secondary metabolite [49,50,51]. However, until now, no study has evaluated the impact of nucleotide polymorphism of such key genes on the biosynthesis of *F. graminearum* secondary metabolites under different conditions, in planta or in vitro.

In this work, we analyzed the genome of *F. graminearum* strains displaying contrasting aggressiveness on bread wheat, their regular host in fields. First, we approximated the repertoire of the *F. graminearum* genes and analyzed their functional characteristics to qualify the intra-specific diversity. Then, we evaluated the contribution of this genomic diversity to the contrasting aggressiveness of the strains, with a particular emphasis on the putative secreted proteins, which are assumed to be involved in host–pathogen interaction. In addition, the impact of mutations in BGCs on the production of secondary metabolites and their relationship with metabolomic profiles was also assessed. These analyses generate new datasets allowing the development of a descriptive model of the complete genome of *F. graminearum*, providing access to a multitude of candidate secreted proteins potentially involved in the pathogenicity of *F. graminearum* and establishing a solid database for further targeted genomic and genetic research.

## 2. Results

### 2.1. Whole Genome Sequencing, Assembly and Annotation

We report the genome sequencing of eight European *F. graminearum* strains, including four French, three Italian and one German isolate (Supplementary Table S1). More than 23,849,853 reads were produced for each of the six strains sequenced by Illumina HiSeq and more than 854,603 subreads with an average length of 7 kb were generated using the PacBio Sequel for the two other strains. The size of the assembled genomes ranged from 36.28 to 37.13 Mb, with a GC content of approximately 48%, as expected for this species. The N50 value of these assemblies ranged between 0.51 and 2.28 Mb, illustrating a reliable contiguity quality. The detailed assembly statistics for the genome of all isolates are summarized in Table 1. 

The completeness of the assembled genomes reached from 97.8% to 99.5% of 3725 core Sordariomycete genes (Supplementary Table S2), with a total number of gene models ranging from 12,729 to 13,316. The cumulative length of the total number of genes accounted for more than 50% of the total length, and the average gene length ranged from 1648 to 1783 bp. In each genome, more than 80% of all annotated genes displayed an Annotation Edit Distance (AED) score between 0 and 0.25, showing high consistency between annotation and overlaps of the ESTs, the mRNA-Seq data and the protein homologies used as evidence. The functional annotation showed that more than 8300 proteins (approximately 65% of the predicted whole proteome) in each strain had known functions, of which more than 64% harbored GO terms (Table 1 and Supplementary Tables S3–S10). The most abundant Pfam domain was the transcription factor “Zn(2)-Cys(6) binuclear cluster” (PF00172), followed by Major Facilitator Superfamily (MFS) transporter (PF07690) with more than 257 and 226 proteins respectively, for each strain. 

### 2.2. Pangenome Analysis and Phylogenomic Relationships of F. graminearum Strains

**Figure 1:**
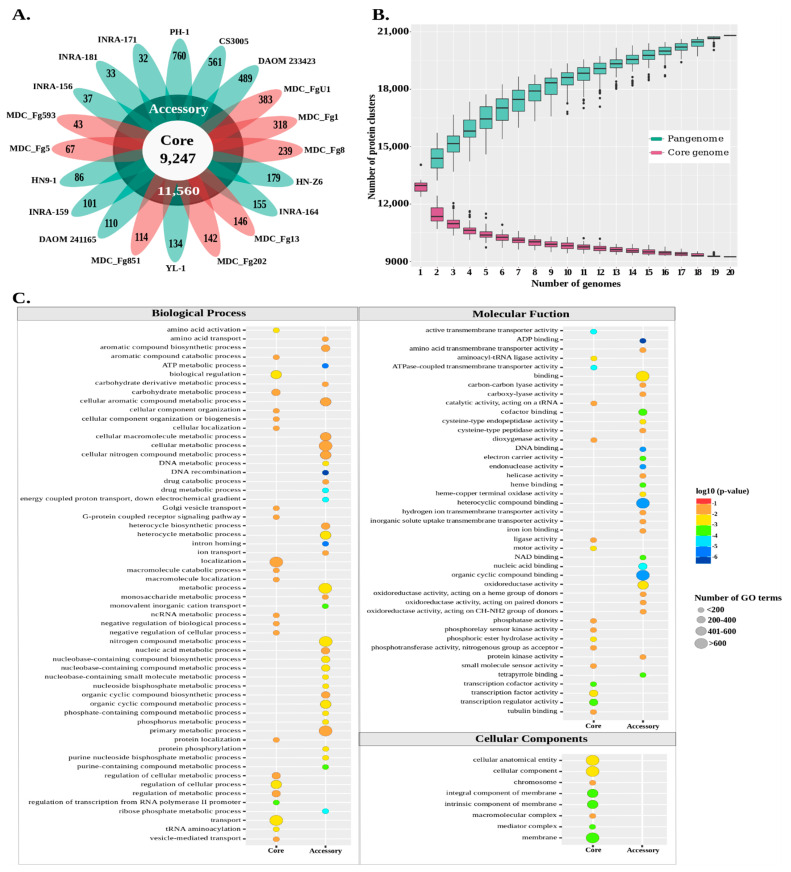
Structure and function of the *F. graminearum* pangenome. (**A**) The flower plot displays the number of core proteins (in the center) and accessory proteins (in the annulus), including the singletons (in the petals), encoded by the genome of 20 *F. graminearum* strains. (**B**) The *F. graminearum* pangenome accumulation curve is depicted with turquoise boxplots, indicating the number of accessory proteins discovered with the sequential addition of new genomes. The purple boxplots illustrate the number of core proteins discovered with the sequential addition of new genomes. (**C**) The density plot depicts the functional enrichment of the core and accessory *F. graminearum* genomes. Enriched GO terms belonging to biological processes, molecular functions and cellular components are presented. The color of the circle represents the statistical significance of the enriched GO terms. The size of the circles represents the number of occurrences of the corresponding GO term.

In order to characterize the intra-species genomic diversity of *F. graminearum* and approximate its complete repertoire of genes, we constructed a pangenome by clustering the protein sets encoded in twenty *F. graminearum* genomes, including the eight from this study and twelve other genomes publicly available (Supplementary Table S1). The clustering parameters were adjusted according to Plissonneau et al. [52] to obtain the most significant homology clusters (Supplementary Table S11). A total of 259,881 proteins were pooled into an open pangenome of 20,807 non-redundant orthologous proteins, of which 9247 (44%) were conserved in the 20 genomes and defined the core genome (Figure 1A, Supplementary Table S12). Nearly 75% (i.e., 6871) of the predicted proteins encoded by the core genome harbored known functions. The accessory genome accounted for 11,560 (56%) non-redundant predicted proteins, of which 7431 (64%) were shared in at least 2 genomes while 4129 (36%) were found in only 1 genome (singleton proteins). Only 34% (i.e., 3958) of the accessory genome had assigned functions. The number of singleton proteins across all genomes ranged from 32 for the INRA-171 strain to 760 for the PH-1 strain (Figure 1A). Among the eight strains of this study, the MDC_FgU1, MDC_Fg1 and MDC_Fg8 ones contained the largest number of singleton proteins, with 383, 318 and 239 respectively, while the MDC_Fg593 and MDC_Fg5 held the lowest number, with 43 and 67 singleton proteins, respectively. In addition to the non-redundant predicted proteins, only 150 redundant paralog proteins were found among the 20 genomes of *F. graminearum* (Supplementary Table S13). The gene accumulation curves show that the numbers of the core genes decreased continually with the addition of new strains (Figure 1B), as expected when sampling more diverging genomes of a species. In contrast, the increasing number of strains systematically yielded novel genes, demonstrating the open state of the pangenome of *F. graminearum*. The core genome was significantly enriched in fundamental processes, including the following GO terms: “biological regulation”, “regulation of cellular process”, “localization” and “transport” (Figure 1C). At the molecular function scale, the core genome was enriched with proteins mainly acting in “transcription regulator activity”, “small molecule sensor activity”, “phosphatase activity” and “phosphotransferase activity”. The accessory genome displayed significant enrichment in proteins involved in “metabolic process”, “binding”, “oxidoreductase activity”, “protein kinase activity” and other catalytic activity. In addition, the cellular component GO terms were enriched only in the core genome (Figure 1C).

**Figure 2:**
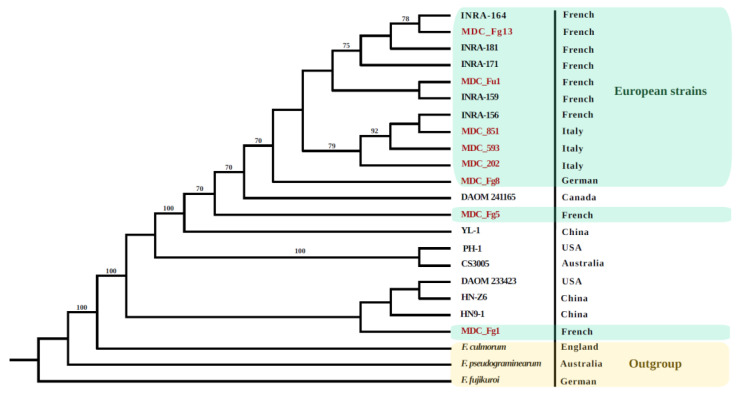
Phylogenetic relationship of 20 *F. graminearum* strains from different geographic origins. A Maximum likelihood bootstrapped cladogram was generated from 7640 single-copy protein sequences found in the 20 *F. graminearum* strains and from 3 other species of the *Fusarium* genus used as an outgroup (*F. pseudograminearum*, *F. culmorum* and *F. Fujikuroi*). Numbers at nodes indicate a bootstrap value greater than 70%, performed with 1000 replications. The country of origin is shown on the right. Names of the 8 strains characterized in this study are shown in red.

The phylogenomic relationships were calculated using the protein sequence homologies of the core genome of the twenty *F. graminearum* genomes and three others of the genus *Fusarium* (*F. pseudograminearum*, *F. culmorum* and *F. fujikuroi*) used as out-groups. Based on 7640 single-copy orthologs, a close relationship between the European genomes of *F. graminearum* was observed, including 7 French, 3 Italian and the German one (Figure 2). The two French MDC_Fg1 and MDC_Fg5 genomes belonged to distinct clades compared to the European genomes. In addition, the non-European *F. graminearum* genomes scattered in the phylogenomic tree. For instance, among the three Chinese genomes, HN-Z6 and HN9-1 have been grouped with the American genome DAOM_233423, while the other American genome, PH-1, was closely related to the Australian strain, CS3005. This analysis also showed that, based on these data, the species *F. graminearum* was more closely related to *F. culmorum* than to *F. pseudograminearum*.

### 2.3. Distribution and Analysis of Genetic Variants in the Eight F. graminearum Genomes

**Figure 3:**
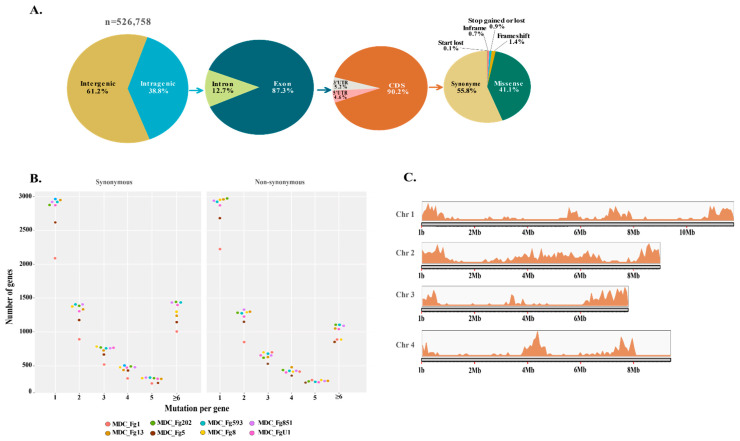
Genome-wide genetic variant distributions. (**A**) Pie charts represent the percentage distribution of the effects of mutations in different genomic regions (inter- and intra-genic), including different types of identified mutations in coding sequences. All the effects expected from the mutations identified in the eight *F. graminearum* genomes, relative to the PH-1 reference sequence, are combined. (**B**) Count distribution of synonymous and non-synonymous mutations per gene is depicted for the eight *F. graminearum* strains. The colored dots correspond to each of the eight genomes. (**C**) Density and localization of genes with a high rate of non-synonymous mutations are depicted along the four *F. graminearum* chromosomes. Only genes with at least six non-synonymous mutations and exhibiting a non-synonymous/(synonymous + non-synonymous) ratio greater than 0.55 were included. The size of the chromosomes is in Mb, in 2 Mb increments.

As a whole, 84% to 98% of the reads produced for the eight genomes were accurately mapped to the reference genome PH-1 (RRES v5.0), covering from 66.72- to 124.56-fold (Supplementary Table S14). Genetic variants were preferentially searched for SNPs and small InDels (less than 50 bp). A total of 353,055 highly confident variant sites differentiated the eight genomes, including 334,087 SNPs (95%) and 18,968 InDels (5%), with an average transition to transversion mutation ratio of 2.57. The number of variants per genome ranged from 108,634 to 145,405 for SNPs and from 4948 to 6568 for InDels (Supplementary Table S14), which is consistent with previous reports [16,53]. The distribution of the expected effects of genetic variants in genomic regions has been studied. The annotation of the 353,055 variant sites predicted nearly 527,000 functional effects. Of these, 61.2% were distributed in the intergenic regions (including the downstream and upstream variants) and 38.8% in the intragenic regions (Figure 3A). Among intragenic variant effects, nearly 87.5% were found in the exonic regions, with more than half (55.8%) having a synonymous effect and 42.1% a non-synonymous one (41.1% were missense effects, 0.9% produced a gain or a loss of stop codon and 0.1% produced a loss of start codon). The remaining 2.1% included both in-frame deletions (deletion of one or several codons) and frameshifts. The number of mutations per gene in each strain showed that MDC_Fg1 systematically displayed the lowest number of genes, with at least one synonymous mutation, followed by the MDC_Fg5 strain (Figure 3B). A similar ranking was observed for the number of genes containing 1 to 2 non-synonymous mutations. 

Considering the 8 genomes, a total of 1128 genes accumulate more than 6 non-synonymous mutations and display a ratio of non-synonymous mutation on the total number of synonymous + non-synonymous mutations higher than 0.6 (Supplementary Table S15). About 46% of these genes (*n* = 522) displayed known functions, including transcription factors, cytochrome P450, protein kinases and enzymes involved in the secondary metabolism. Their distribution along the four chromosomes of the PH-1 reference genome showed that they are preferentially located in the proximal regions of the telomeres and in the central regions of the chromosomes. Chromosome 2 had the highest number of these genes despite being shorter than chromosomes 1 and 4 (Figure 3C). 

Among the range of identified mutations, we noticed 1283 genes displaying loss-of-function (LOF) mutations (Supplementary Table S16). The number of LOF genes for each genome ranged from 396 (in the MDC_Fg1 strain) to 582 (in the MDC_FgU1) (Supplementary Figure S1) and only 6% (*n* = 79) were shared in the 8 genomes, while more than 30 genes were specifically lost in each genome.

### 2.4. Accumulation of Mutations in Secondary Metabolite Gene Clusters and Relationships with Metabolomic Profiles

We examined genetic variants in the 67 known secondary metabolite gene clusters characterized in *F. graminearum* [30]. The trichothecene biosynthetic gene cluster (BGC), encoding enzymes and proteins directly involved in the virulence on wheat tissues [54], displayed the highest number of variants compared to all the other BGCs. The trichothecene BGCs are defined by 15 *TRI* genes: 12 are grouped in chromosome 2, while 3 are distributed on 3 other chromosomes (Supplementary Table S17). A total of 1316 and 1165 different mutations was found in the intra- and inter-genic regions of trichothecene BGCs, respectively (Supplementary Table S18). The two Italian genomes MDC_Fg202 and MDC_Fg593 displayed a particularly high number of genetic variants, gathering 96% of the total intragenic mutations. These corresponded to 306 non-synonymous and 6 frameshift mutations in MDC_Fg593 and 224 non-synonymous and 7 frameshifts in MDC_Fg202. The *TRI12* (coding for the MFS transporter involved in the efflux of trichothecene) and the *TRI8* (coding for the C-3 esterase [55]) genes accumulate more non-synonymous mutations than other *TRI* genes (68 in *TRI12* and 66 in *TRI8* found in MDC_Fg593, and 55 in *TRI12* and 72 in *TRI8* found in MDC_Fg202). The other six genomes harbored a smaller number of non-synonymous mutations in the *TRI* genes, with only two in MDC_Fg1 and MDC_Fg5, thirteen in MDC_Fg8 and MDC_Fg851 and sixteen in MDC_Fg13 and MDC_FgU1. In addition, a total of 210 LOF mutations were identified in 107 genes belonging to 47 secondary metabolite BGCs, including the trichothecene, the fusarin, the fusaristatin, the butanolide and the zearalenone BGCs (Supplementary Table S19). 

**Figure 4:**
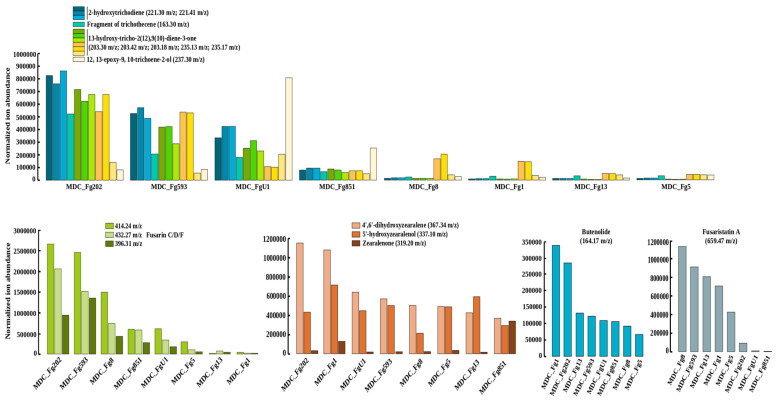
Abundances of selected metabolites in the eight *F. graminearum* strains. Ion abundances were derived from extracted ion chromatography (XIC) profiles obtained from mass spectrometry analysis (LC/MS) of the eight strains grown on PDA medium. Different detected ions corresponding to trichothecene biosynthesis (see Supplementary Figure S2 for details, mass-to-charge ratio (*m/z*): 163.30, 203.30, 203.42, 203.18 235.13, 235.17, 221.30, 221.41, 237.30), fusarin isoforms (retention time 19.8 min and *m/z* values of 396.31 for M-2xH2O+H, 414.24 for M-H2O+H, 432.27 for M + H), zearalenone and its intermediates (retention times 21.1, 23.5 and 34.3 min with m/z values of 319.20, 337.10, 367.34, respectively), Butenolide (retention time 2.9 min with *m/z* value of 164.17) and Fusaristatin A (retention time 27.9 min and *m/z* value of 659.47) are reported.

Given the high rate of genetic variants in these secondary metabolite BGCs, we profiled the metabolite content of each of the eight *F. graminearum* strains, using a shot-gun metabolomics approach. Metabolite profiling shows substantial differences in metabolite abundance, in the eight strains, measured by ion abundances (Figure 4). MDC_FgU1 produced the most 12,13-epoxy-9,10-trichoene-2-ol. The two Italian strains (MDC_Fg202 and MDC_Fg593), that accumulate a high number of genetic variants in the trichothecene BGC, displayed the highest abundance of the trichothecene intermediates (Figure 4 and Supplementary Figure S2). These two strains also displayed the highest amount of fusarins. Regarding the range of LOF mutations, no obvious links were evidenced between enzymes with truncated sequences and the expected metabolite they are supposed to produce. For instance, intermediate abundances of fusarin C were detected in the MDC_Fg8 strain when it was observed to harbor a LOF mutation in the fus1 gene (FGSG_07798), one of the pivotal enzymes required for the synthesis of this metabolite. Another discrepancy has been noted for zearalenone found at the highest abundance in the MDC_Fg851 strain, where this strain includes a LOF mutation in a gene responsible for its production. A non-ribosomal peptide synthetase (NRPS7, FGSG_08209) belonging to the gene cluster that mediates the biosynthesis of fusaristatin A, and a transporter (FGSG_08084) belonging to the butenolide BGC, were found with a LOF mutation in MDC_Fg5 and MDC_FgU1 respectively, while high abundance of these metabolites was found in these two strains.

### 2.5. Characterization of the Aggressiveness of the Eight F. graminearum Strains on Wheat Spikes

**Figure 5:**
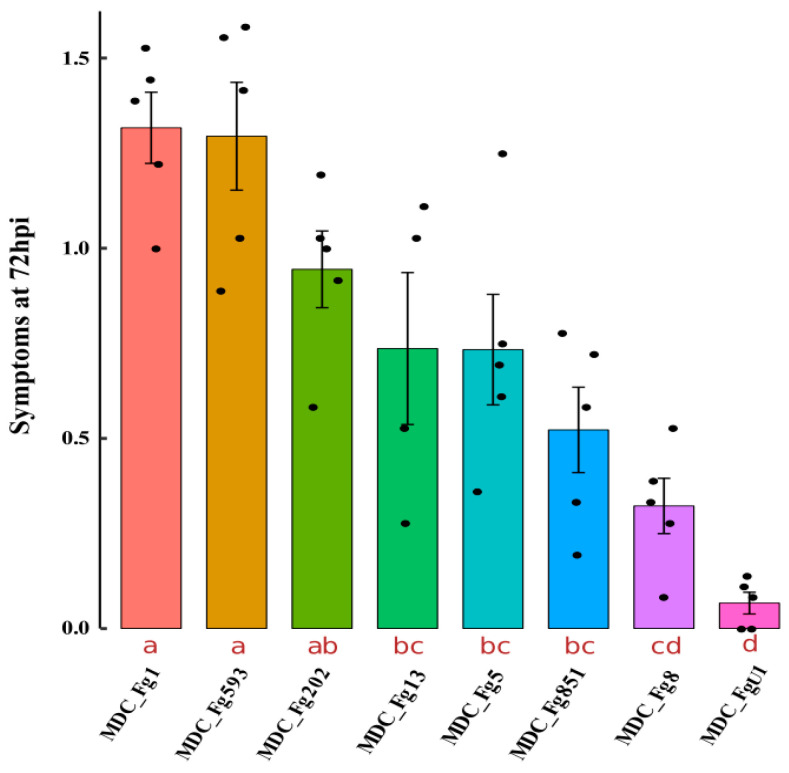
Symptom severity produced by the eight *F. graminearum* strains in bread wheat spikes during the FHB disease progress. The eight strains of *F. graminearum* were inoculated on the Recital wheat cultivar and symptoms were monitored at 72 hpi according the scoring described by Fabre et al. [56]. The *Y*-axis represents the symptom severity scale ranging from 0 to a maximum of 4. The error bars are the means ± SE of five biological replicates, and each replicate was characterized by the average value (represented as black dots) computed from six spikelets of three inoculated spikes. The different letters indicate significant differences at *p* ≤ 0.05 (Duncan’s multiple range test for post-hoc ANOVA mean separation).

Our previous work has shown that a rating of symptoms during the first 72 h on the FHB-susceptible winter wheat Recital clearly differentiated three different strains of *F. graminearum* [17]. The aggressiveness of the eight *F. graminearum* strains was thus evaluated according to the same procedure by rating inoculated spikelets at 72 hpi, specifically on this wheat cultivar (Figure 5). The two MDC_Fg1 and MDC_Fg593 strains systematically induced the most intense symptoms compared to the MDC_Fg202, MDC_Fg13, MDC_Fg5 and MDC_Fg851 which produced intermediate symptoms on the wheat spikelets. The German MDC_Fg8 and the French MDC_FgU1 were the least aggressive strains, especially the latter one that induced very weak symptoms at 72 hpi. 

### 2.6. Comparative Analysis of Secreted Protein Contents in F. graminearum Strains of Contrasting Aggressiveness

**Figure 6:**
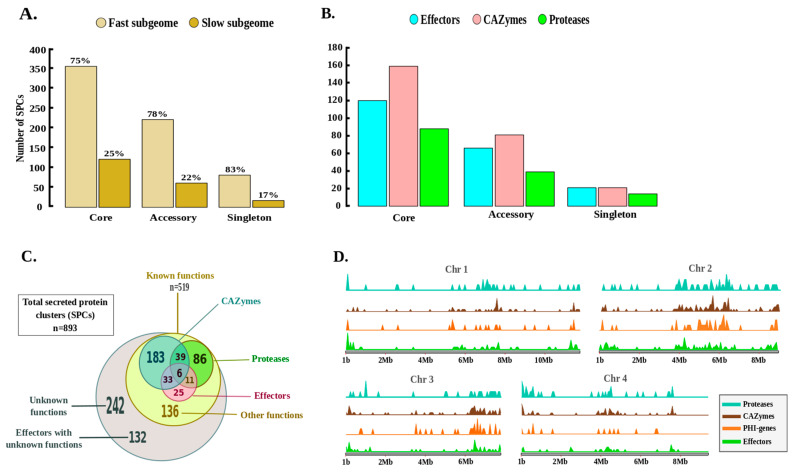
Characterization of the *F. graminearum* pan-secretome. (**A**) Categorization of putative secreted protein clusters (SPCs) of the eight *F. graminearum* strains into core, accessory and singleton secreted proteins. In each of the three categories, the percentage of SPCs assigned to the fast and slow sub-genome is reported. (**B**) The bar graph depicts the number of SPCs encoding CAZymes, proteases and putative proteinaceous effectors (according to EffectorP v2.0) in each of the three pan-secretome categories. (**C**) The Venn diagram depicts the number of SPCs displaying one or more labels (among CAZyme, protease or effector), as well as those with no predicted functions. (**D**) Density and localization of the genes encoding secreted CAZymes, secreted proteases, secreted effectors and genes potentially involved in the host–pathogen interaction (according to PHI-base) along the four *F. graminearum* chromosomes. The size of the chromosomes is in Mb, in 2 Mb increments.

In each strain, about 5% of the theoretical proteins harbored secretory characteristics. The pan-secretome included 893 non-redundant secreted protein clusters (SPCs), of which 476 (53%) belonged to the core secretome (SPCs found in the 8 strains), 292 (33%) to the accessory secretome (SPCs found in 2 to 7 strains) and 125 (14%) were specific to 1 strain (Figure 6A, Supplementary Table S20). More than three-quarters of the pan-secretome belonged to the fast sub-genome, including 75%, 78% and 83% of the core, accessory and singleton secreted proteins, respectively.

Nearly 60% of the SPCs (*n* = 519) displayed known functions (Figure 6C), half of which (*n* = 261) corresponded to putative secreted CAZymes (Table S20), including 110 glycoside hydrolases (GH). CAZymes also included 59 enzymes belonging to auxiliary activities (AA) and 51 carbohydrate esterases (CE). In addition, 40% (*n* = 207) were putative secreted effectors and 142 SPCs corresponded to putative secreted proteases, including 82 serine peptidases and 32 metallo-peptidases. Overall, nearly 15% of all SPCs belonged to at least two functional categories of CAZymes, proteases and secreted effectors (Figure 6C), and nearly 60% of the CAZymes, proteases and putative effectors are part of the core secretome, and are thus expected to be conserved in the eight strains (Figure 6B). 

To go further into the possible links between secreted protein roles and fungal aggressiveness, the pathogen–host interaction (PHI) database was also queried. A total of 108 known PHI genes were retrieved in the 8 genomes, among which two-thirds belonged to the core secretome (Supplementary Table S19). Overall, 50 proteins were assigned to the “unaffected pathogenicity” group, 38 to the “reduced virulence” group, 4 to the “avirulence plant determinant” and 1 from the “loss of pathogenicity” group, while 15 were part of the “mixed outcome” category (i.e., found in more than one group). The unique secreted protein belonging to the “loss of pathogenicity” group corresponds to a superoxide dismutase [57] detected in four strains of strong or intermediate aggressiveness (MDC_Fg1, FG001_06131; MDC_Fg593, FG593_06654; MDC_Fg13, FG013_08273; MDC_Fg851, FG851_03677). Likewise, among the four proteins belonging to the “avirulence plant determinant” group, three were found in all strains, while one containing a LysM domain was observed only in the aggressive strain MDC_Fg593 (FG593_11638) (Supplementary Table S20). The density and localization of genes encoding these SPCs, in particular those encoding CAZymes, proteases, effectors and PHI genes, along the four chromosomes of PH-1 evidenced the presence of highly enriched and compartmentalized genomic regions, with conserved distributions in each chromosome (Figure 6D). The proximal regions of the telomeres and the central regions of the four chromosomes especially harbored these SPCs, and a particular accumulation was found in chromosome 2 compared to the other three chromosomes.

LOF mutations in SPCs were further investigated. A total of 162 secreted proteins belonging to 77 SPCs harbored LOF mutations (Supplementary Table S20). The least aggressive strain MDC_FgU1 displayed the highest number of truncated secreted proteins (LOF mutations in 36 proteins) compared to the other seven strains. Among these, 13 were expected to remain intact in all the other genomes, including a phosphoesterase (FU001_11254), three CAZymes of the PL4, AA5 and GH145 families (FU001_02749, FU001_08346 and FU001_07816, respectively), two serine peptidases (FU001_06394, FU001_06650) and two secreted proteins belonging to both CE10 CAZyme and peptidase groups (FU001_08354 and FU001_08976).

### 2.7. Predicted Subcellular Localization of Secreted Proteins in the Plant Cell

**Figure 7:**
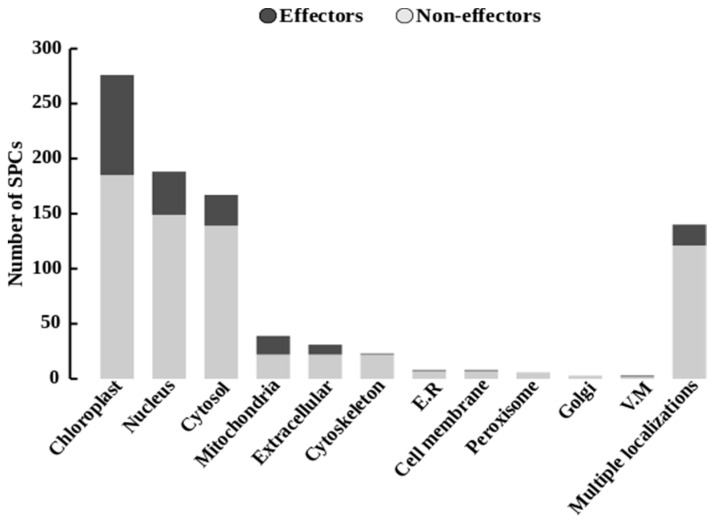
Expected subcellular targeting of SPCs in the plant cell. Bar graphs represent the predicted subcellular localization of putative secreted proteins (light gray), including the secreted putative effectors (dark gray). The pipeline used for the prediction of the secretion characteristics is described in Supplementary Figure S3.

Prediction of the subcellular localization of all SPCs on mature proteins (without the signal peptide sequence) demonstrated the targeting of three major subcellular compartments (Figure 7, Supplementary Table S20). The chloroplast target was predicted for 277 (31%) SPCs, of which 91 were putative effectors. Only 26 of these displayed known functional domains, including fourteen CAZymes (seven CE, three CBM, two GH, one AA and one PL), five proteases (two serine peptidases, two metallo-peptidases and one peptidase inhibitor), two Killer toxin (kp4) and one Ecp2 effector. The plant nucleus localization was identified for 188 (21%) SPCs, including 39 putative effectors, 10 of which displayed significant homology with CAZymes (six GH, two PL, one AA and one CBM), five with proteases (two serine peptidases, two metallo-peptidases and one cysteine-peptidase), one coding for a fungal lipase, one cerato-platanin and one for Killer toxin (kp4). The cytosol target was predicted for 167 SPCs (18.7%), including 28 putative effectors, of which half display known functions. A multiple localization signature (SPCs expected to target more than one cell compartment) was evidenced in 140 SPCs (15.7%), including 19 putative effectors. The 13.6% remaining SPCs were expected to target other plant compartments, such as the mitochondria (4.4%), the apoplasm (3.47%), the cytoskeleton (2.57%), the endoplasmic reticulum (0.9%), the plasma membrane (0.9%), the peroxisomes (0.7%), the Golgi apparatus (0.33%) or the vacuolar membranes (0.33%).

## 3. Discussion

### 3.1. Structure and Functional Diversity at the Intra-Species Scale of F. graminearum

Previous comparative genomic analyses of *F. graminearum* mainly focused on the genome diversity within the *F. graminearum* species complex (FGSC), particularly on shared and absent genes as compared to a single reference genome [58,59,60,61]. In the present study, an intra-specific comparative genomic analysis was conducted to study the variability within the genomes of *F. graminearum* species. Walkowiak et al. [59] identified a list of 15,297 genes from 10 FGSC genomes, including 12% accessory genes (genes absent in at least one genome). Our study extends the pangenome of *F. graminearum* to approximate its full repertoire of genes based on twelve already reported genomes of different geographical origins and complemented with eight newly sequenced genomes of European strains. Our results showed that *F. graminearum* contains a significantly larger gene repertoire than that previously known from the reference genome PH-1 [25,26], with 20,807 non-redundant proteins. Less than half (45%) characterized all the genomes. The size of this core genome appears to reach a constant estimate and it is unlikely that the addition of other new genomes could significantly extensively reduce its size. The phylogeny, based on the shared proteins, shows that the European genomes are mostly grouped and closely related, as compared to non-European genomes, suggesting that they belong to a related population. Only the two French genomes, MDC_Fg1 and MDC_Fg5, belonged to separate clades. This could be due to the high rate of singleton SNPs which was only found in these two genomes, as well as a lower number of mutations per gene as compared to the other genomes of our study. Besides these core proteins, the high proportion of accessory predicted proteins suggests that the pangenome of *F. graminearum* is of the open type, indicating that the inclusion of other annotated genomes is likely to increase the size of the gene repertoire. This indicates that the strains harbor a high level of unique genomic diversity, which may reflect the ability of *F. graminearum* to survive in different environments [62]. Reaching 55% of the pangenome, the number of accessory and specific genes emphasizes the occurrence of potentially redundant functions. Given that our pangenome analysis and previous analyses of the PH-1 reference genome revealed little evidence of paralogy [24,60], it is unlikely that functional redundancy has evolved due to gene duplications. The action of the repetition-induced mutation system (RIP), which operates in *F. graminearum* [63], is known to protect the fungal genome against the activity of transposable elements and also limits the genome evolution by duplication of genes [64,65]. This could also argue for the low number of repeated sequences compared to other phytopathogenic species [66,67].

Considering the eight genomes we sequenced, a substantial fraction of the proteins (*n* = 1283) potentially lost their function because of LOF mutations such as frameshifts, gains of stop codon, losses of start codon, or splice acceptors and donors. Although these numerous truncated proteins are unlikely to perform a biological function identical to the intact corresponding protein, no direct link has been evidenced with strain development, pathogenicity or virulence through experimental tests, suggesting that functional redundancy compensation processes could occur, as already reported in *Botrytis cinerea* [68]. Besides functional redundancy and genomic truncations, the analysis of non-synonymous mutation rates indicated that 1129 other genes may be under diverse selection. As other phytopathogens, *F. graminearum* is known to have a “two-speed” genome with comparable sizes and number of genes [28,29]. More than 90% of the genes showing an excess rate of non-synonymous mutations were over-represented in the fast sub-genome, emphasizing that this sub-genome evolves faster than its slow counterpart. In addition, these genes are preferably grouped together and located in the proximal regions of the telomeres of the four chromosomes, or in the most central regions of the three chromosomes (1, 2 and 4). This is consistent with previous work showing links between the SNP density and high recombination frequency occurring in these chromosomal areas [16,24]. Our results support the idea that interstitial regions with high mutation rates result from ancestral chromosome fusions that merged ancestral telomeres [24,58,69]. 

Functional analysis showed that trichothecene BGC particularly held high rates of non-synonymous mutations, especially in the *Tri8* and *Tri12* genes. *Tri8* is a C3-esterase able to influence the synthesis of different forms of trichothecenes (both 15-acetyl-DON and T-2 toxin), while the *Tri12* gene is supposed to be involved in trichothecene production and efflux [55,70]. As a whole, the links between genetic variations of *TRI* genes and the aggressiveness of *F. Graminearum* strains are not clear, emphasizing that trichothecenes are only part of the molecular determinants of pathogenicity. At the strain level, only the two Italian genomes, MDC_Fg202 and MDC_Fg593, systematically accumulated a high rate of genetic variation in these two aforementioned genes, as well as a remarkable number of variants found in the intra- and inter-genic regions of trichothecene BGC, while they are among the most aggressive strains. These results partly corroborate the in vitro metabolomic profiling observed, in which only MDC_Fg202 and MDC_Fg593 exhibited an increased abundance of the trichothecene precursors, while little difference can be observed in the trichothecene products, suggesting that the accumulation of mutations identified in these *TRI* genes could play an important role in the accumulation of trichothecene precursors. Many previous studies have already shown that genetic variants can affect metabolite production in *A. fumigatus*, *A. nidulans* and *B. cinerea* [71,72,73,74]. However, in our study, no direct link was observed between other metabolites and severe effects of genetic variants, such as LOF mutations affecting the genes responsible for the production of these metabolites. This suggests that the accumulation of numerous mutations in BGCs could have a wider impact on metabolite production than LOF mutations in a single gene of the pathway. This also suggests the existence of a possible functional redundancy/compensation in the genes required for metabolite biosynthesis.

### 3.2. Links between the Genetic Variations in F. graminearum Secretomes and the Strain Aggressiveness in Wheat Tissues

In this analysis, we studied the refined secretomes of eight *F. graminearum* strains with high contrasting aggressiveness on a winter wheat genotype. Brown et al. [43] already predicted a secretome in the reference PH1 strain, indicating that 4.2% of the total genes encode classical secreted proteins. Here, approximately 5% of the total gene number harbored secretion characteristics (classical and non-classical secreted proteins) in each of the eight genomes. This proportion seems to be a regular content which should be observed in any refined secretome of *F. graminearum*. Overall, 893 SPCs defined the pan-secretome of the 8 strains and are considered to be putative secreted proteins. The functional analysis revealed 374 (42%) SPCs of unknown function, of which more than a third (*n* = 132) were predicted as putative secreted effectors. This exemplifies that unknown functions of secreted proteins and putative fungal effectors are usual characteristics in *F. graminearum,* as in other filamentous plant pathogens [41,75,76,77]. The prediction of their subcellular localization in the host cell demonstrated that the chloroplast could be a primary target of *F. graminearum* effectors, followed by the plant nucleus. Although functional characterizations of these proteins might be necessary to identify their intracellular localization and their role in the infection process, this clearly corroborates previous dual-proteomics on the FHB disease in bread wheat that identified a close relationship between chloroplast adjustments and the accumulation of *F. graminearum* proteins harboring the features of a chloroplast transit peptide [17,56,78]. Among SPCs with known functions, more than 70% are closely linked to plant/pathogen interaction and pathogenicity, including CAZymes, proteases and PHI genes. This finding emphasizes that *F. graminearum* aggressiveness depends on an arsenal of secreted proteins. Since they were mostly located in regions known as chromosomal recombination hotspots [24,43], this could therefore facilitate the occurrence of genetic variations to adapt with changes in the host responses. More than two-thirds of all SPCs belonged to the fast sub-genome, where genes also rapidly evolve to facilitate swift adaptation and infection responses [28,29]. *F. graminearum* colonizes the surface of wheat via runner hyphae (RH) and develops infection cushions (IC) which are essential for the penetration of the fungus into the plant cuticle [19,79,80]. Compared to mycelium grown in complete medium, both RH and IC have already shown that 982 genes were systematically upregulated during initial infection of wheat floral tissues [19]. Of these genes, 194 were included in our pan-secrectome, of which 74% belonged to the core secretome and 88% were located in the fast sub-genome, emphasizing their pivotal role in the infection process. Moreover, in another previous study, we identified 321 proteins of the MDC_Fg1 strain that were significantly regulated in planta during a 96 hpi period [56]. Among these, nearly three-quarters (73%) belonged to the slow sub-genome, suggesting that this slow sub-genome includes the necessary drivers of the disease progression through the accumulation of proteins with critical roles in pathogenicity.

Given the large differences in aggressiveness in the eight strains, we sought the possible links with the content of their secretomes, including with known secreted proteins involved in pathogenicity. For instance, 23 secreted proteins proved to be specific to the aggressive MDC_Fg1 strain, including 7 proteins predicted as proteinaceous effectors, with only 1 (FG001_05220) assigned to a known function encoding α/β-hydrolase and displaying a putative nuclear localization signal. Although a number of other specific secreted proteins have been identified in the most aggressive strains, their connections with aggressiveness require further functional analyses to be validated. In contrast, most of the putative secreted proteins linked to pathogenicity, including the PHI-genes, CAZymes, proteases and effectors candidates, were found in the core secretome, thus systematically shared by the eight strains. This corroborates previous results demonstrating that the three French, MDC_Fg1, MDC_Fg13 and MDC_FgU1, strains synthesize in planta very similar proteins at 72 hpi, and that the differences in aggressiveness could rather be due to differences in protein accumulation [17]. Consequently, no obvious qualitative and quantitative relationship can be defined between the SPCs linked to pathogenicity and the strain aggressiveness, and this high level of conservation in the eight secretomes suggests that *F. graminearum* secreted proteins can be involved in both the ability to survive as a pathogen and as a saprophyte [43]. In spite of a few qualitative differences in the SPCs among the eight strains, we nevertheless detected a number of truncated secreted proteins by LOF mutations, specifically in the least aggressive MDC_FgU1 strain. Among these SPCs, proteins with crucial roles in the hydrolysis of polysaccharide components of the cell wall of plants and in the degradation of host defense proteins such as GH145, PL4, AA5, serine peptidases and carboxylesterase type B were specifically truncated in MDC_FgU1, while they were intact in the other strains. This further suggests that different *F. graminearum* strains are characterized by a core SPC signature, with, however, marginal differences that may partly lead to discrepancies in aggressiveness. 

## 4. Materials and Methods

### 4.1. Fusarium graminearum Isolates and Strain Aggressiveness Rating 

Eight European *F. graminearum* isolates were reported in this study, and all isolates were able to produce the metabolite deoxynivalenol as well as induce FHB on bread wheat. Of these isolates, four were collected from France (MDC_Fg1, MDC_Fg5, MDC_Fg13 and MDC_FgU1) in field trials between 2010 and 2012 (Supplementary Table S1). MDC_Fg1, MDC_Fg13 and MDC_FgU1 have already been characterized in a previous study dealing with fungal proteomics during FHB [17]. Three other isolates were originated from Sardinia (Italy: MDC_Fg202, MDC_Fg593 and MDC_Fg851), and they were collected in 2018. The MDC_Fg8 isolate was collected from Germany and was obtained from Dr. T. Miedaner [81] and extensively characterized within a number of reports [54,79,80,81,82,83,84]. For each isolate, the preparation of the *F. graminearum* inocula was performed as described by Fabre et al. [56] to obtain a concentration of 10,000 spores per mL.

Aggressiveness of the eight *F. graminearum* strains was evaluated through symptom monitoring on the winter wheat cultivar Recital, as described by Fabre et al. [17,56]. After sowing seeds, plantlets underwent an 8-week vernalization at 4 °C, then they were transferred in a growth cabinet with optimal conditions to allow synchronized flowering (16 h daylight at 20 °C, 8 h darkness at 18 °C and 80% relative humidity). A total of 40 homogeneous plants were prepared in individual 4 L pots and divided into 5 complete randomized blocks, surrounded by 30 additional plants to control any edge effect. In each block, one plant was used to monitor the aggressiveness of each of the eight strains, which corresponds to five different replicates. At the mid-anthesis stage, the eight strains were individually inoculated into randomly selected plants in each block by depositing 10 μL of inoculum in the floral cavity of six contiguous spikelets located in the middle zone of three synchronized spikes per plant. These represented a total of 18 spikelets per plant (90 measurements per strain) that were individually scored for the symptom dynamics at 72 h after the inoculation (hpi). This duration was chosen on the basis of our previous studies [17,56,78] demonstrating a turning point in the FHB progress at this time, enabling to discriminate strains of contrasted aggressiveness. Along with the forty infected plants, five additional ones were inoculated with water and were used as a control, and no symptoms were observed on these plants.

### 4.2. Genome Sequencing and Assembly

The fungal strains were grown on potato dextrose broth and the genomic DNA was prepared from the mycelium using a NucleoBond kit according to the manufacturer’s instructions (Macherey-Nagel, Düren, Germany). *F. graminearum* MDC_Fg5, MDC_Fg8, MDC_Fg13, MDC_202, MDC_Fg593 and MDC_Fg851 were sequenced using the Illumina HiSeq platform. MDC_Fg1 and MDC_FgU1 were sequenced by the PacBio SMRT Sequel platform. A 150 bp paired-end library was prepared following the manufacturer’s instructions. After quality control, the percentage of reads with a Q20 and Q30 score ranged from 95 to 98.5 and 90 to 97, respectively. The reads were preprocessed with Trimmomatic v.36 [85] using default settings. Over 87% of preprocessed reads were used for de novo assembly with ABySS v2.0.0 [86] using three k-mer sizes (41, 96 and 128). 

The SMRT Bell library was prepared using a DNA template Prep 1.0 kit (Pacific Biosciences, CA, USA) according to the manufacturer’s protocol. A size selection of 12 kb was realized by using the BluePippin system (Sage Scientific, Beverly, MA, USA) according to the protocol “20 kb Template Preparation Using BluePippin Size-Selection System”. The raw PacBio Sequel subreads were filtered and assembled de novo using the Hierarchical Genome Assembly Process (HGAP4) implemented in SMRT Link v5.0 [87] using default settings, which keeps only subreads with read quality (rq) of ≥0.7. The completeness of the assembled genomes was assessed by the presence of 3725 single-copy conserved genes from the Sordariomyceta lineage using Benchmarking Universal Single Copy Orthologs Version 3 (BUSCO v3) [88].

### 4.3. Gene Prediction and Genome Annotation

The assembled genomes were annotated using the MAKER2 Annotation Pipeline [89], with a combination of evidence-based methods (transcriptome data and homology with known proteomes) and ab initio gene prediction. In brief, four samples of transcriptomic RNAseq reads of methylation-depressed *F. graminearum* obtained from NCBI (Run accessions: SRR999640, SRR999641, SRR999642 and SRR999643) were assembled by rnaSPAdes v3.13.0 [90] and used as evidence, with 21,355 expressed sequence tags (EST) of *F. graminearum* PH-1 (downloaded from NCBI).

Protein evidence from UniProtKB (update-2018_09) supplemented with the proteomes of *F. graminearum* PH-1, *F. pseudograminearum*, *F. culmorum*, *F. fujikuroi*, *F. oxysporum* and *F. verticillioides* retrieved from Ensembl Fungi (update-40) were also used for homology research. Two different ab initio gene annotation programs have been trained for use with MAKER2. SNAP v2013-02-16 [91] was used with transcriptome sequences and Augustus v3.2.1 [92] was used with *F. graminearum* gene models. The repetitive elements of the whole genomes were masked using the library of all *Fusarium* repeated elements of the Repbase database (update-01.02.2017) via RepeatMasker v4.0.7 [93]. The same annotation method was used for ten other *F. graminearum* genomes with only published raw data, including five French, three Chinese, one American and one Canadian (Supplementary Table S1).

### 4.4. Pangenome Construction and GO Enrichment Analysis

The eight genomes of our study and twelve other genomes of *F. graminearum*, including five French (INRA-156, INRA-159, INRA-164, INRA-171 and INRA-181), three Chinese (YL-1, HN9-1 and HN-Z6), two American (PH-1 and DAOM_233423), one Canadian (DAOM_241165) and one Australian (CS3005) (Supplementary Table S1) were used for the pangenome construction. Reciprocal best BLAST hits [94,95] were performed to identify the set of orthologous genes using the Proteinortho v5.16b [96]. After comparing several values of identity (30% to 90%) and coverage (60% to 90%), based on conservation of the pangenome size [52], proteins with an identity score above 50% and a sequence coverage above 70% (e-value below 1 × 10^−5^) were deemed as significant hits. Core genome and pangenome size curves were generated using proteinortho_curves [96] with 100 iterations. To analyze the GO enrichment, functional annotation was performed using InterProScan v5.13-52.0 [97], including the Pfam database families and the gene ontology (GO) classification. The enrichment of GO terms was evaluated using the Python package GOATOOLS v0.8.2 [98] and the go-basic.obo database (version 2020-02-06). The *p*-values used to assess the significant enrichment of the GO terms were calculated based on a Fisher’s exact test with propagation of the parent term and correction for the false discovery rate (FDR, *p* < 0.05).

### 4.5. Phylogenomic Analysis

To construct a phylogeny of the twenty genomes of *F. graminearum* described below, completed by three other genomes of the genus *Fusarium* (*F. pseudograminearum*, *F. culmorum* and *F. fujikuroi*) used as outgroups, we used protein sequences found in exactly one copy in all the representatives of the twenty-three genomes based on a cluster analysis. The identified single-copy orthologs were aligned using MAFFT v7.402 [99] and poorly aligned regions were removed by using Gblocks v0.91b [100] with default settings. Maximum likelihood (ML) analysis was performed with RaxML v8.1.20 [101] using the “–f a” option and the PROTGAMMLG amino acid substitution matrix model with 1000 bootstrap replicates. The phylogenetic tree was visualized using FigTree v1.4.4 (http://tree.bio.ed.ac.uk/software/figtree (accessed on 7 July 2020)).

### 4.6. Analysis of Secreted Proteins and Prediction of Their Subcellular Localization in the Plant Cell

The eight annotated proteomes of *F. graminearum* of this study were used for the prediction of secretomes downstream by performing the pipeline described in Supplementary Figure S3. The secretory signal peptide was predicted using SignalP v4.1 [102]. Protein sequences lacking the signal peptide were used by SecretomeP v1.0 [103] for the prediction of non-classical secreted proteins. After that, the signal sequences approved by SignalP and SecretomeP were analyzed by TargetP v1.1b [104] to further remove mitochondrial proteins. The sets of proteins were examined for the presence of a transmembrane domain using TMHMM v2.0c [105], and only proteins without a transmembrane domain or one transmembrane domain in the N-terminal signal peptide were kept. WoLFPSORT v0.2 [106] was used to select the proteins with an extracellular destination with a score greater than 17, as mentioned by Brown et al. [43]. Lastly, only sequences without GPI (glycosylphosphatidylinositol) anchors predicted by PredGPI [107] in these extracellular proteins were retained as candidates for secreted proteins. The subcellular localization of secreted proteins in plant cells were predicted using a combination of three tools, LOCALIZER [108] running in “effector mode” and WoLFPSORT [106] running in “plant mode” after removing the fungal secretion signal, while nuclear localization signals (NLS) were confirmed using the cNLS Mapper [109].

### 4.7. Pathogenicity-Related Genes’ Prediction

The refined secretomes were screened for Carbohydrate Active enZymes (CAZymes, Appendix A) using Hmmscan in HMMER v3.1b2 (http://hmmer.org/ (accessed on 16 July 2020)) and the dbCAN HMM profile database v6 [110]. The outputs were further processed by the hmmscan-parser script provided by dbCAN to select significant matches. Putative peptidases and peptidase inhibitors were predicted using the MEROPS database (update-12.1) [111]. To identify proteins potentially associated with pathogenicity, the BLASTP search was performed against the Pathogen–Host Interaction database v4.5 (PHI-base) [112,113]. BLASTP searches against the MEROPS database and PHI-base were used with an e-value threshold of 10−5 with the best hits and an identity greater than 40%. Besides the PHI genes, CAZymes and secreted peptidases, we also searched for proteinaceous effectors by characterizing the secreted proteins using the pipeline described above (Supplementary Table S3) and by adding a search with EffectorP v2.0 to distinguish the secreted proteinaceous effectors in the predicted fungal secretomes [114].

To determine the location in the chromosome and the distribution of genes coding for putative secreted proteins linked to pathogenicity (CAZymes, peptidases, PHI-genes and effectors), we aligned these coding genes with the PH-1 reference genome RRES v5.0 using GMAP v2020-06-30 [115] using the default settings. The gene density plots on the four chromosomes were generated using karyoploteR [116].

### 4.8. Variant Calling and Analyses

The filtered reads were mapped on the PH-1 reference genome RRES v5.0 using BWA-MEM v0.7.17-r1188 (with option -M) [117] for the data generated by Illumina HiSeq and Minimap v2.12-r847 [118] for those released by PacBio SMRT Sequel. The mapping quality was assessed by Qualimap v2.2.1 [119]. 

The six BAM files (results from Illumina HiSeq data mapping) were sorted, and duplicated reads were removed by Picard SortSam and MarkDuplicates v1.119, respectively (http://broadinstitute.github.io/picard/ (accessed on 7 September 2020)). Positions of insertions/deletions (InDels) were recalibrated using the RealignerTargetCreator and the IndelRealigner modules of the Genome Analysis Toolkit (GATK, version 3.8-1-0-gf15c1c3ef) [120] with standard parameters. The single nucleotide polymorphisms (SNPs) and short InDels were named using the GATK Unified Genotyper in haploid mode. To obtain high-quality variants, we used the GATK VariantFiltration to perform hard filtering based on quality cut-offs following the GATK best practice recommendations. For SNPs, we filtered out: QD < 2.0||FS > 60.0||QUAL < 250||MQ < 40.0||SOR > 3.0||MappingQualityRankSum < −12.5||ReadPosRankSum < −8.0. For InDels, we filtered out: QD < 2.0||FS > 200.0||SOR > 10.0||QUAL < 250||ReadPosRankSum < −20. Only insertions and deletions shorter than or equal to 50 bp were kept. 

To identify variants from the two other Bam files (PacBio Sequel subread mapping results), we first generated mpileup files using SAMtools, v1.8, with -Q0 -e 5 -C50 –B options [121]. The result was used as input to VarScan, version 2.3.9 [122], with the “mpileup2snp” and “mpileup2indel” functions to identify SNPs and short indels respectively, with a minimum average coverage of 10, a minimum variant allele frequency of 0.2, minimum frequency to call homozygote of 0.85 and a *p*-value threshold of 0.05.

The final call set of the eight genomes was annotated and their impact was predicted using SnpEff v 4.3t [123] with the –ud 500 and –lof options. Variant effects were parsed with SnpSift v4.3t [124] and vcftools v0.1.16 [125]. The SnpEff databases were built locally using annotations of the reference genome PH-1 RRES v5.0 obtained in GTF format from Ensembl Fungi (update-40). The SnpEff database was used to annotate SNPs and InDels with putative functional effects according to the categories defined in the SnpEff manual (http://snpeff.sourceforge.net/SnpEff_manual.html (accessed on 27 November 2020). Genes were accepted with an excess of mutational mutations if they contained at least more than six non-synonymous mutations and showed a ratio of non-synonymous mutations to the total number of synonymous and non-synonymous mutations greater than 0.6, according to Laurent et al. [16].

### 4.9. Secondary Metabolite Analysis

Strains were grown on PDA (Potato Dextrose Agar, Difco, 1.5% agar) plates for eight days at 25 °C. For metabolomics analysis, four plates of each strain were frozen, then thawed and extracted with 50 mL ethyl acetate (EtOAc). The mixture was shaken at 200 rpm and ambient temperature for 48 h, then filtered through filter paper. The ethyl acetate phase was separated from the water and dried in vacuo. Each extract was prepared to 40 mg/mL in 1:1 ACN:methanol (MeOH) for LC/MS analysis. Each sample was analyzed in duplicate on an Agilent 1100 series LC/MS platform. Positive mode ionization was used to detect metabolites. Datasets were exported from Agilent’s Chemstation software as .netCDF files and imported into MZmine v2.3 [126]. Peak picking was performed with established protocols [48]. Mass detection was centroid with 1.7 × 10^4^ minimum height. Chromatogram building was limited to peaks greater than 0.1 min with 0.1 mass-to-charge ratio (*m/z*) tolerance and 1.8 × 10^4^ minimum height. Chromatogram deconvolution utilized local minimum search, with 92–99% threshold, 40 min search range, 1% relative height, 1.9 × 10^4^ minimum abs. height, 1 minimum ratio of top/edge and peak duration from 0.10 to 10.00 min. All treatments were then aligned with a tolerance of 0.05 *m/z* and relative retention time tolerance of 3.0 (%). Peak finder gap filling was performed with 25% intensity tolerance and 0.1 *m/z* tolerance. Duplicated peaks were combined with a tolerance of 0.5 *m/z* and 0.3 min. The normalized data were processed using MetaboAnalyst software v4.0 [127]. Data were log-transformed and scaled to unit variance for a multivariate modeling approach to check for variability based on overall metabolite profiles. Data were quantitated to scalar graphs for *Fusarium* compounds by their extracted ion detection relative to each of the eight *F. graminearum* isolates. 

## 5. Conclusions

This study expanded our understanding of the intra-species genomic diversity of *F. graminearum* and provided new data on its genetic content and its relationship with the FHB disease. First, this work showed that a single reference genome sequence depicting less than 68% of a pangenome does not fully reflect the gene content of any strain belonging to this species due to the high degree of observed genomic variation. This emphasizes that switching from a reference genome to a reference pangenome, gathering the dispensable or accessory genomic regions we described in this study, could arguably refine a wide range of genomics or transcriptomics analyses, and further improve variant calling and the accurate quantification of gene expression. Lastly, although further analyses are required to fill the gap between comparative genomics and strain pathogenicity, all of these data substantially improve our knowledge about the genomic variations of *F. graminearum* strains displaying contrasting aggressiveness. They provide pivotal clues to further decipher the molecular determinants of the FHB disease and bring to the forefront a wealth of candidate secreted proteins potentially involved in the pathogenicity of *F. graminearum*, including a number of new candidate effectors that will guide future research.

## Figures and Tables

**Table 1:** Genome assembly and annotation statistics of the eight *F. graminearum* genomes of this study.

Strains	MDC_Fg1	MDC_Fg5	MDC_Fg8	MDC_Fg13	MDC_Fg202	MDC_Fg851	MDC_Fg593	MDC_FgU1
Assembly statistics								
Genome size (Mb)	36.80	36.56	36.28	36.75	37.13	36.75	36.45	37.01
k-mer for ABySS assembly	Na ^a^	128	41	96	96	96	96	Na ^a^
Total number of contigs	96	297	1.599	283	292	278	203	139
Total number of contigs > 1 Kb	95	131	1.327	176	185	155	140	139
Max contig size (Mb)	3.41	7.74	0.27	2.33	1.87	1.98	2.88	1.67
N50 (Mb)	1.65	2.28	0.51	0.79	0.77	0.65	1.02	0.65
G + C content (%)	47.97	48.16	48.29	48.32	48.28	48.32	48.33	48
BUSCO completeness (%)	98.63	99.41	98.90	99.46	99.38	99.41	99.41	97.85
Gene annotation								
Number of gene models	13,166	12,790	12,993	13,297	13,189	13,316	12,729	13,014
Total gene length (Mb)	21.69	22.80	22.63	21.93	23.14	22.02	21.66	22.85
Mean gene length (bp)	1648	1783	1742	1650	1755	1654	1702	1756
Coding region in genome (%)	50.7	52.6	52.8	51.6	52.6	51.8	51.3	51.6
Number of exons	36,189	38,400	38,475	36,415	39,181	36,670	35,721	38,824
Total exon length (Mb)	19.65	20.29	20.18	19.96	20.59	20.03	19.72	20.16
Mean exons per gene	2.7	3.0	3.0	2.7	3.0	2.8	2.8	3.0
Mean exon length	543	528	525	548	526	546	552	519
Number of introns	23,023	25,610	25,482	23,118	25,992	23,354	22,992	25,810
Total intron length (Mb)	2.09	2.57	2.50	2.03	2.60	2.04	2.0	2.74
Mean intron per gene	1.7	2.0	1.9	1.7	1.9	1.7	1.8	2.0
Mean intron length (bp)	91	100	98	88	100	87	87	106
Protein functions								
Genes with IPR/Pfam annotations	8536	8345	8409	8653	8504	8675	8634	8400
Genes with GO annotations	5501	5449	5469	5570	5531	5584	5558	5501
Secreted proteins	659	653	650	692	667	692	670	662

^a^ Assembled by HGAP4.

## Data Availability

The Whole Genome Shotgun (WGS) projects; raw data, assembled and annotated genomes of the eight strains of this study were deposited in ENA/GenBank/DDBJ under the accession numbers listed in Table S1 (BioProject accession no. PRJEB27611).

## Supplementary Materials

The following are available online at https://www.mdpi.com/article/10.3390/ijms22126257/s1.

## Abbreviations

GH, glycoside hydrolases; CE, carbohydrate esterases; AA, auxiliary activities; GT: glycosyltransferases, CBM, carbohydrate-binding modules; PL, polysaccharide lyases; S, serine; M, metallo; C, cystein; T: threonine; A, aspartate, N, aspargine; G, glutamate; I, inhibitors.

## Appendix A

We compared the global distribution of CAZymes and proteolytic enzymes in *F. graminearum* with 15 other fungal genomes of the Sordariomycetes class and one genome from the Dothideomycetes class (used as an outgroup) with different lifestyles. We observed that *F. graminearum* contained a larger number of genes coding for CAZymes compared to other pathogenic fungi, such as *Verticillium dahliae* (necrotrophic), *Gaeumannomyces graminis* (necrotrophic), *Verticillium alfalfae* (necrotrophic) and *Magnaporthe oryzae* (hemibiotrophic) (Supplementary Figure S4a). In the eight genomes of *F. graminearum* sequenced in this study, an average of 654 proteins distributed in 139 CAZyme families were systemically predicted, of which 66 (48%) were conserved in all the analyzed fungal species. In addition, four CAZyme families were found in the *F. graminearum* genomes only, GH14 (β-amylase), GH73 (lysozyme), GT61 (β-1,2-xylosyl-transferase) and CBM47 (Fucose-binding activity).

Regarding proteases, our results revealed that the *F. graminearum* had the fourth highest number of predicted representatives, just after *Nectria haematococca* (Necrotrophic), *Fusarium oxysporum* (Necrotrophic) and *Magnaporthe oryzae* (Hemibiotrophic) (Supplementary Figure S4b), with an average of 742 proteases belonging to 122 families and 38 protease inhibitors from 16 families. Serine peptidases were observed as the largest category, followed by metalloproteases and cysteine peptidases. A similar trend was observed in the other analyzed fungal species. Furthermore, the comparative analysis identified only one protease family specifically found in the genomes of *F. graminearum* (M26, IgA1-specific metallopeptidase).
