# Supplementary material for: Comparative Genomics of Eight Fusarium graminearum Strains with Contrasting Aggressiveness Reveals an Expanded Open Pangenome and Extended Effector Content Signatures
Source: Int J Mol Sci. 2021 Jun 10;22(12):6257. doi: 10.3390/ijms22126257 (PMC8230406; doi:10.3390/ijms22126257)
Supplement: Supplementary file 1 [file ijms-22-06257-s001.zip › Figure S2.pdf]

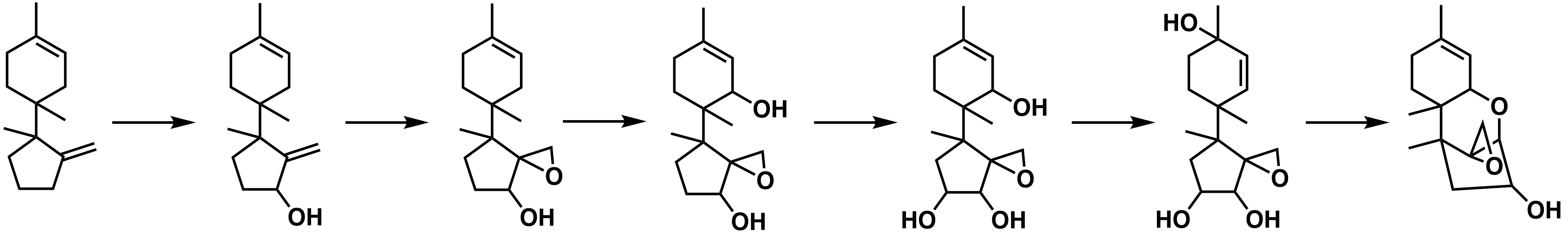

Compound	MW	$[M+H]^+$
trichodiene	MW = 204.36	$[M+H]^+$ = 205
2-hydroxytrichodiene	MW = 220.36	$[M+H]^+$ = 221
12,13-epoxy-9,10-trichoene-2-ol	MW = 236.36	$[M+H]^+$ = 237
isotroichodiol	MW = 252.53	$[M+H]^+$ = 253
isotrichotriol	MW = 268.35	$[M+H]^+$ = 269
trichotriol	MW = 268.35	$[M+H]^+$ = 269
isotrichodermol	MW = 250.34	$[M+H]^+$ = 251

221.30mz/12.23min	2-Hydroxytrichodiene
221.30mz/12.23min	2-Hydroxytrichodiene
221.41mz/12.21min	2-Hydroxytrichodiene
163.30mz/12.22min	fragment of 2-hydroxytrichodiene
203.30mz/12.25min	13 Hydroxy-tricho-2(12),9(10)-diene-3-one
203.42mz/12.25min	13 Hydroxy-tricho-2(12),9(10)-diene-3-one
203.18mz/12.24min	13 Hydroxy-tricho-2(12),9(10)-diene-3-one
235.13mz/12.81min	13 Hydroxy-tricho-2(12),9(10)-diene-3-one
235.17mz/12.03min	13 Hydroxy-tricho-2(12),9(10)-diene-3-one
235.17mz/12.08min	13 Hydroxy-tricho-2(12),9(10)-diene-3-one
237.30mz/13.21min	12,13-Epoxy-9,10-trichoene-2-ol

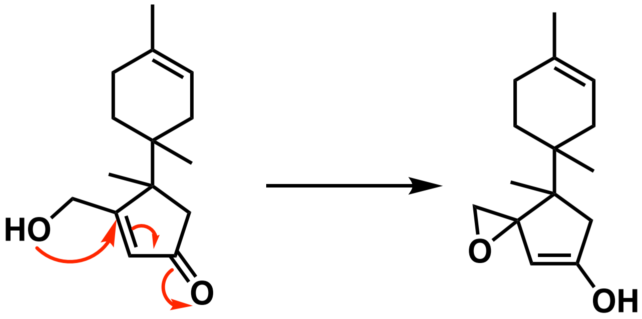

13-hydroxy-tricho-2(12),9(10)-diene-3-one

MW = 234.34
$[M+H]^+$ = 235
$[M-CH_2OH]^+$ = 203

MW = 234.34
$[M+H]^+$ = 235
