# Supplementary figures and images for: Comparative Genomics of Eight Fusarium graminearum Strains with Contrasting Aggressiveness Reveals an Expanded Open Pangenome and Extended Effector Content Signatures
Source: Int J Mol Sci. 2021 Jun 10;22(12):6257. doi: 10.3390/ijms22126257 (PMC8230406; doi:10.3390/ijms22126257)

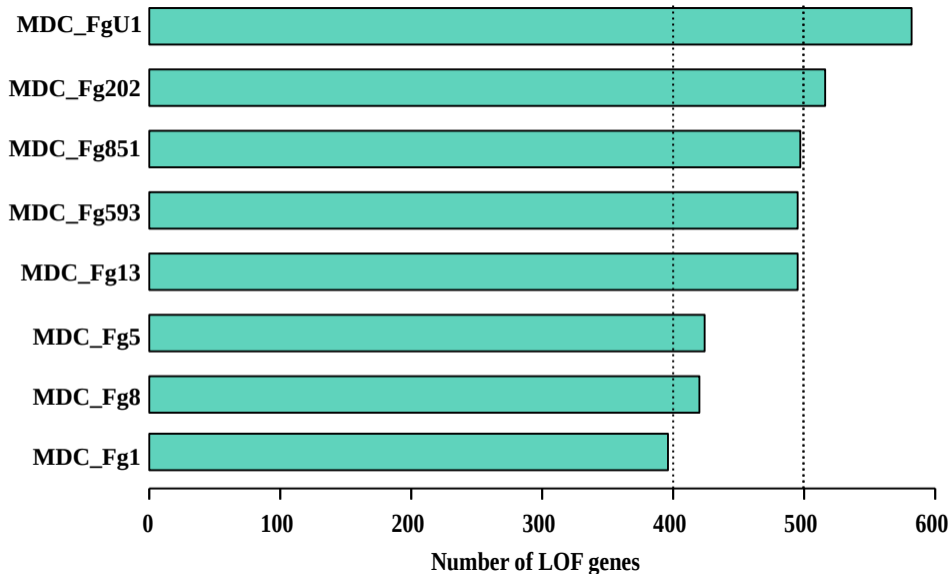

Supplement: Supplementary file 1 [file ijms-22-06257-s001.zip › Figure S1.pdf]

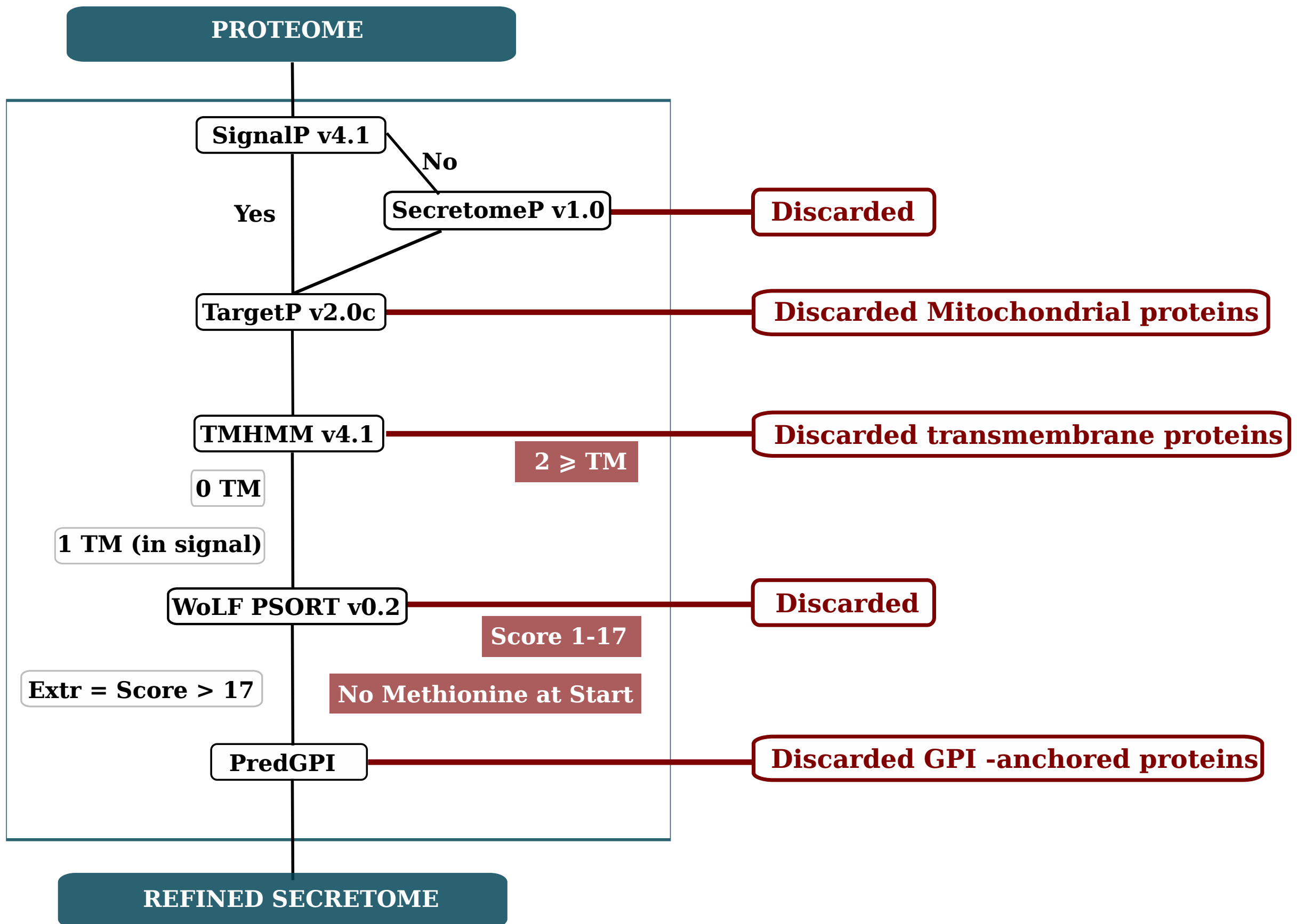

Supplement: Supplementary file 1 [file ijms-22-06257-s001.zip › Figure S3.pdf]

Fig.S2

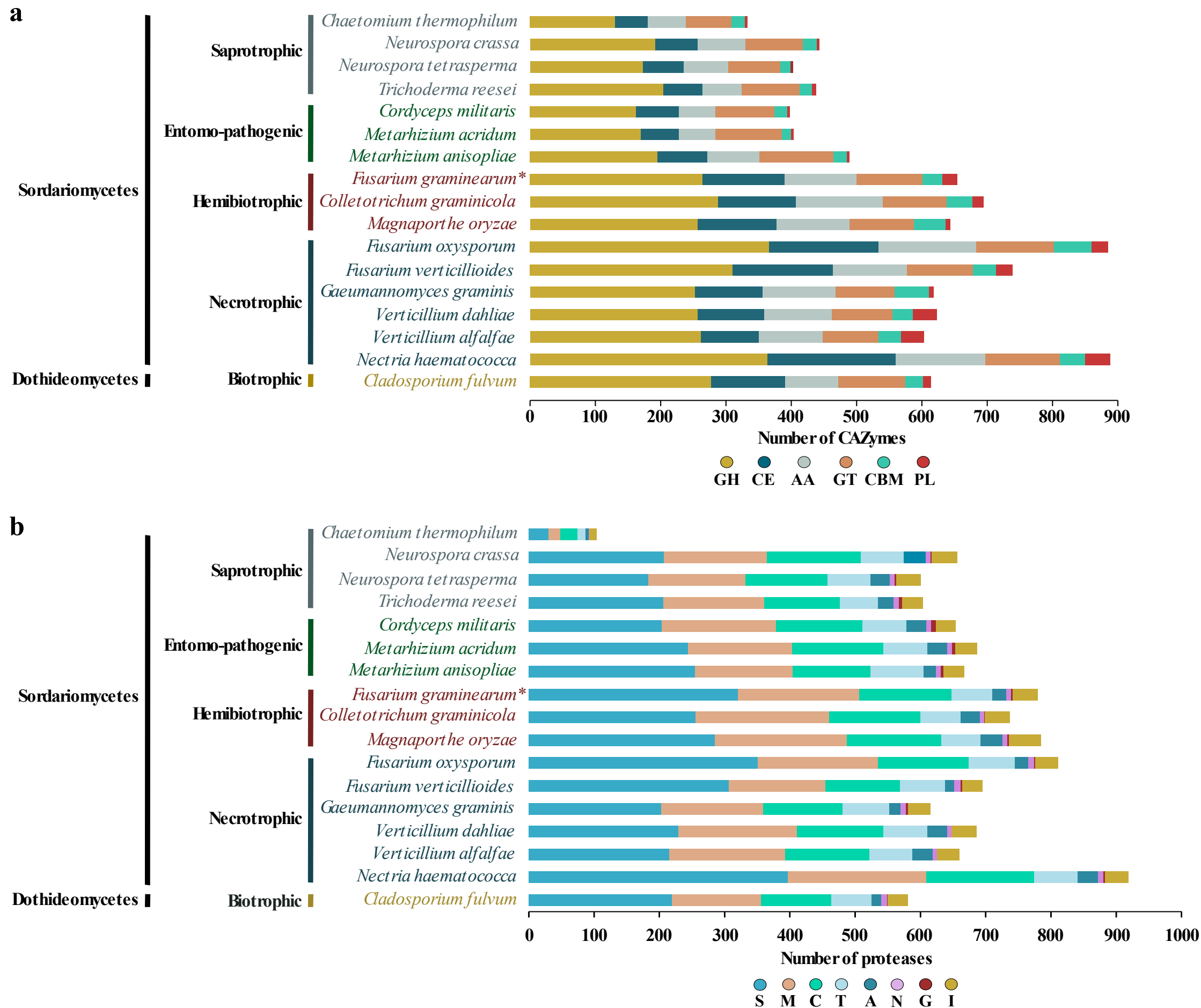

Supplement: Supplementary file 1 [file ijms-22-06257-s001.zip › Figure S4.pdf]
